# Perceived teacher autonomy support for adolescents’ reading achievement: The mediation roles of control-value appraisals and emotions

**DOI:** 10.3389/fpsyg.2022.959461

**Published:** 2022-08-29

**Authors:** Meishu Wang, Jie Hu

**Affiliations:** Department of Linguistics, School of International Studies, Institute of Asian Civilizations, Global Competency Center, Zhejiang University, Hangzhou, China

**Keywords:** teacher autonomy support, control-value appraisals, achievement emotions, multiple mediation modeling, PISA reading

## Abstract

Analyzing students’ internal cognitive-motivational appraisals and achievement emotions is of pivotal importance for educational outcomes and student individual wellbeing, yet little is shown about their associations with teacher autonomy support. This study investigates the relationship between perceived teacher autonomy support and reading achievement by addressing mediating influences of control and value-related constructs, i.e., reading self-efficacy, meaning in life, and reading enjoyment. Multiple mediation modeling was adopted to test the proposed model with carrying out a total of 12,058 students from 361 schools in China in the Programme for International Student Assessment (PISA) 2018 reading database. The results elucidated that student-perceived teacher autonomy support is significantly positively related to adolescents’ reading outcomes by fostering internal control and value appraisals and academic enjoyment. These current findings confirm the positive correlation between teacher autonomy support and adolescents’ motivational and emotional factors, providing significant practical implications for educators to adopt various teaching strategies to enhance adolescents’ self-efficacy, intrinsic values, and positive emotions.

## Introduction

Reading ability is perceived as the pivotal skills for successful integration into modern society (OECD, 2019). Although there are a wide range of factors that might influence adolescents’ reading competence, teacher autonomy support have indicated to be the most crucial exogenous factors in learning processes and reading achievement (e.g., Cheon et al., 2018; Quin et al., 2018). Autonomy supportive teachers might relinquish much control of students’ learning process, adopt structure and motivating style by providing multiple solution pathways with students for internalizing and externalizing problems (Vansteenkiste et al., 2012; Bureau et al., 2022a). A cornucopia of studies have confirmed the notion that teacher autonomy support is positively linked to students’ personal characteristics (Bureau et al., 2022a), emotional wellbeing (Gilbert et al., 2021), dogged perseverance (Reeve and Cheon, 2021), active learning engagement (Olivier et al., 2020), and educational performance (Guay et al., 2019), whereas teacher highly controlling is linked to students’ frustration of psychological needs, disengagement and low learning outcomes (Aelterman et al., 2019).

According to control-value theory (CVT), as an essential component of learning environment, teacher autonomy support is closely related to students’ appraisals (Pekrun et al., 2014), including perceived control, e.g., self-efficacy and perceived intrinsic value, e.g., meaning in life, which are acted as the antecedents of achievement emotions (e.g., Wigfield et al., 2015; Roorda et al., 2017). Self-efficacy refers to the belief in an individual capacity to successfully complete a task or execute a specific behavior in a specific domain (Bandura, 1995), reflecting students’ perceived control (Skinner, 1996; Pekrun et al., 2014). Additionally, as “the primary motivational force” (Frankl, 1963, p. 121), meaning in life serves as an intrinsic value (Siwek et al., 2017), involves the cognition and behavioral processes to meet the desire, spurring people to search for significance and purposes of their lives (Steger et al., 2008). Specifically, students who perceived more teacher autonomy support tend to have strong cognitive appraisals and show positive emotions during learning activities (Putwain et al., 2021).

Therefore, understanding the mechanism behind teacher autonomy support and whether it has a negative or positive influence on control-value appraisals and emotions, provides valuable insights into adolescents’ wellbeing and educational attainment (Ryan and Deci, 2017; Putwain et al., 2021). Despite recent studies shed light on the relationship between teacher autonomy support and student psychological factors, limited evidence has probed into the correlations among teacher autonomy support, students’ cognitive appraisals and emotional factors. Nonetheless, studies exploring their correlations mostly concentrated on math or science achievement (e.g., Wang et al., 2017), physical activities (e.g., Zimmermann et al., 2021), scarce studies related these factors to students’ reading performance and elucidated that how these relationships can operate in secondary school students in reading activities.

This study aims to fill these lacunas underlying the interrelationships between student-perceived teacher autonomy support, appraisals, achievement emotions, and reading achievement in a sample of 15-year-old students. Specifically, this study examines how control-value appraisals in tandem with subsequent achievement emotions, and their mediating role between teacher autonomy support and reading achievement. Pekrun’s control-value theory (CVT) is adopted as theoretical framework in this study. This theory offers an integrative theoretical perspective concerning the non-linear relationship between learning situations and educational outcomes (e.g., Wigfield et al., 2015; Roorda et al., 2017). It highlights that the correlations between person and environment are condensed in various appraisals (e.g., self-efficacy, meaning in life, etc.) and achievement emotions (e.g., Pekrun, 2006; Pekrun and Stephens, 2010; Daniels and Stupnisky, 2012). As such, this theory is consistent with the present study.

## Literature review

### Teacher autonomy support and reading achievement

Student behavioral, cognitive, and academic development are intimately associated with a supportive learning environment where teachers provide tangible help, guidance, and explanations to support student learning (e.g., Hughes et al., 2012; Pekrun et al., 2014; Wentzel, 2016). In autonomy-supportive environment, teachers try to attract students by using inviting language to provide interesting learning activities, detailed explanations and related them to students’ own experience (Wentzel et al., 2010), which offers students a chance to activate their cognition in learning process (Lei et al., 2018; Yu and Hu, 2022). Teachers also allows for students’ independent thinking, expressing their perspectives freely and studying in their own peace (Bureau et al., 2022b; Yu et al., 2022). Student-perceived teacher autonomy support refers to how student convinced of teachers providing helps on their learning (Wentzel, 2016; Zimmermann et al., 2021). The more students perceived teacher autonomy support, the more they gain emotional identification and feel fulfillment of their psychological needs (e.g., Quin et al., 2018; Zimmermann et al., 2021; Yang et al., 2022). Thus, they are more likely to get involved in a wide range of learning activities, make free choices based on their own interests (Lazarides and Buchholz, 2019; Preece and Levy, 2020).

Empirical studies provided evidence on the positive relations between teacher autonomy support and students’ academic achievement. For instance, Förtsch et al. (2016) revealed that teacher autonomy support such as cognitive activation has a significant positive associations with students’ learning outcomes and explain 15% of the variance in academic achievement. In reading context, Olivier et al. (2020) demonstrated that teacher autonomy support might offer students a positive leaning environment, motivate students’ reading engagement and enhance their reading achievement. Similarly, Guay et al. (2019) suggested that teacher autonomy support is a positive predictor of student intrinsic motivation and reading achievement in Grade 1 students. However, Kikas et al. (2016) claimed that higher individualized support is negatively related to reading comprehension skills and reading fluency among primary school students.

In light of the previous research, teacher autonomy support is closely linked to students’ academic achievement and reading achievement. However, the results are inconsistent with both the positive and negative relationships of teacher autonomy support with reading achievement. Apart from that, teacher autonomy support in previous studies was evaluated mainly focused on individual help, while this study focused on the students-perceived teacher autonomy support for the whole class instead of specific people.

### Teacher autonomy support, control-value appraisals, and reading achievement

According to CVT, control-value appraisals refer to one’s competence beliefs, expectancies, and attributional style, and consist of subjective control and subjective values of learning outcomes, which are closely tied with achievement activities (Pekrun and Stephens, 2010). It can be shaped by interactions with learning environment (Pekrun, 2006). Perceived as a pivotal component in learning environment, teacher autonomy support poses an influence on students’ appraisals (Luo et al., 2016). Specifically, the way teachers manage the class and teaching strategies they adopted are supposed to empower students to participant in various tasks and make essential learning choices, which affects students’ perceived control and intrinsic values (e.g., Wang et al., 2017; Zimmermann et al., 2021).

Despite some studies have examined the relationship between teacher autonomy support and students’ appraisals, the internal correlations among student-perceived teacher autonomy support, students’ self-efficacy and meaning in life has not been examined in reading context. Although substantial studies have explored the relationship between self-efficacy and reading achievement, the results are inconsistent. Some studies have reported that self-efficacy is positively related to reading performance. That is, when students have a strong sense of their reading competence, they are more likely to work harder and persevere in these difficult reading activities (Linnenbrink and Pintrich, 2003; Peura et al., 2019a), and attain high reading achievement. For instance, Solheim (2011) demonstrated that reading self-efficacy was significantly positively associated with reading comprehension scores. Similar findings were also obtained by Sewasew and Koester (2019), who presented a reciprocal relationship between self-perception of competence and reading achievement and found a positive association of self-efficacy with relevance to reading achievement. However, Peura et al. (2019b) found that the relationship between self-efficacy and reading outcomes varied based on the level of task specificity and the measurement of academic achievement. They elucidated that specific and intermediate self-efficacy had a positive association with reading outcomes, whereas general self-efficacy was not. Moreover, Carroll and Fox (2017) suggested that high reading self-efficacy might not be closely related to high reading performance.

As an internal value construct, meaning in life involves individuals having beliefs about values and purposes. It illustrates how individuals consistently pursue learning goals despite various challenges and difficulties (Steger et al., 2008; Hill et al., 2016). Individuals who have a higher level of meaning in life are rarely suffering existential frustration and psychological problems (Makola, 2014). Previous evidence reported that having meaning in life makes a difference in one’s cognition and behaviors and is closely linked to positive outcomes, such as self-esteem (Steger et al., 2006) and enjoyment (Steger et al., 2006), and thus produces a significant influence on relevant educational outcomes (e.g., Steger et al., 2008). Browman et al. (2019) suggested that having meaning in life might activate one’s upward mobility and initiative, which might encourage individuals to persevere in pursuing their goals, and is positively related to academic outcomes. Bailey and Phillips (2016) found that college students’ intrinsic motivations such as meaning in life has positive associations with their academic performance. Despite the positive influence of meaning in life on academic outcomes having been confirmed, there are limited studies exploring the relationship between meaning in life and reading performance, not even with teacher autonomy support, meaning in life, and reading achievement. Moreover, limited studies have thoroughly researched the mediated relation of control-value appraisals as a whole and its relationship with teacher autonomy support and reading achievement.

### Teacher autonomy support, reading enjoyment, and reading achievement

As the central role in CVT, achievement emotions refer to the emotions that students experience in ongoing learning activities or testing contexts (Pekrun, 2000; Mercan, 2020). Among multitudinous achievement emotions, enjoyment is commonly explored and has attracted much research attention due to its positive influence on learning (Simonton and Garn, 2020; Zaccoletti et al., 2020), which are significantly associated with cognitive processes, motivational beliefs, and learning environment factors (Pekrun, 2006; Schunk and Usher, 2019). Empirical studies largely support a close relationship between teacher autonomy support and positive achievement emotions, i.e., reading enjoyment (e.g., Lazarides and Buchholz, 2019; Bureau et al., 2022a). As part of reading motivation factors, reading enjoyment involves the enjoyment or satisfaction of curiosity individuals engaged in reading activities (OECD, 2019). Reading enjoyment has been indicated, to be enhanced by teacher stimulation in engagement, guidance, and informational support (Roorda et al., 2017). Additionally, researchers have identified the role of reading enjoyment among multitudinous predictors in reading achievement and have consistently demonstrated a positive connection (e.g., Taboada et al., 2009; Wolters et al., 2014). Such evidence has shown that individuals who enjoy reading, are more likely to read and engaged in a wide range of reading tasks (Taboada et al., 2009). Moreover, students with higher level of reading enjoyment tend to choose more challenging reading texts, take the initiative to apply effective reading strategies, or seek support from others more often than individuals with a lower level of reading enjoyment (e.g., Park, 2011; Lim and Jung, 2019). Thus, students with high reading enjoyment exhibit high reading achievement (Morgan and Fuchs, 2007).

However, previous studies have mostly examined the influence of teacher autonomy support and reading enjoyment on reading outcomes among primary school students, few studies have focused on secondary school students. Therefore, this study will examine the mediating role of reading enjoyment in the relationship between perceived teacher autonomy support and reading achievement among adolescents.

### The relationship between control-value appraisals and reading enjoyment

The CVT delineates that different achievement emotions arise from different control- and value-related constructs (Pekrun, 2006; Daniels and Stupnisky, 2012). Studies have also noted that the combination of positive control and value appraisals are acted as additive predictors of positive emotions, i.e., enjoyment (e.g., Goetz et al., 2012). For instance, Zimmermann et al. (2021) examined the role of appraisals and achievement emotion in leisure-time physical activities. They found that perceived control, i.e., self-efficacy and intrinsic value are statistically significantly positively related to enjoyment in physical activities. Buhr et al. (2019) highlighted that control and value appraisals generate more enjoyment and less boredom in a massive open online course. Putwain et al. (2021) reported that student-perceived control, i.e., perceptions of self-competence beliefs in math and intrinsic value are significantly positively related to their later math enjoyment. Similarly, Wang et al. (2017) confirmed that math self-efficacy and intrinsic value can be significantly negatively related to negative emotions, i.e., boredom in math. Simonton and Garn (2020) claimed that a student who intrinsically believed him- or herself to be competent or enterprising in learning activities and has own intrinsic values tends to experience positive achievement emotions, e.g., feel enjoyment in the learning process.

Based on the previous literature, it can be seen that most of them are focused on other academic contexts, such as mathematics or leisure activities instead of reading achievement. Additionally, most of the previous studies have regarded intrinsic value as a whole without considering the relationship between meaning in life and emotional factors of adolescents specifically. Therefore, this study explored the relationship between control-value appraisals and academic enjoyment by addressing self-efficacy and meaning in life in a reading classroom.

### The present study

Previous theoretical and empirical evidence indicates the potential interrelations between teacher autonomy support and students’ learning outcomes. However, despite considerable studies have been conducted on the relationship between autonomy support of teachers and educational attainment, scarce studies have probed into the reading context, and examined how teacher autonomy support interacts with control-value appraisals, and academic enjoyment and thus related to reading achievement. Even so, the findings are incomprehensive and worthy of up-to-date scrutiny, given the increasingly importance of motivational and emotional factors in adolescents’ individual development. Explicitly, most of the previous studies shed light on primary school students or college students, scarce evidence concerning secondary school students. Additionally, there are relatively few studies using the international large-scale database, i.e., Programme for International Student Assessment (PISA) (2018), to explore the mediating role of control-value appraisals and reading enjoyment in the relationship between the teacher autonomy support and reading outcomes.

**FIGURE 1:**
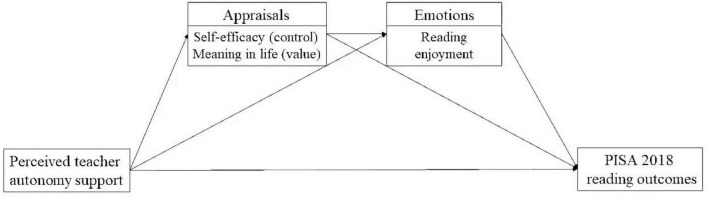
The conceptual framework of the current study.

Therefore, this study expands the previous literature by examining the correlations among student-perceived teacher autonomy support, control- and value-related constructs (i.e., self-efficacy in reading, meaning in life), and achievement emotions (i.e., reading enjoyment) proposed in CVT, adopting a sample of secondary school students in PISA 2018 reading assessment. Figure 1 presents the conceptual framework of this study. Based on the CVT theory, we tested the following hypothesis:

(1) Control-value appraisals (i.e., reading self-efficacy, meaning in life) will be mediated by the student-perceived teacher autonomy support and reading achievement.
(2) Reading enjoyment will be mediated by the student-perceived teacher autonomy support and reading achievement.
(3) Both control-value appraisals (i.e., reading self-efficacy, meaning in life) and reading enjoyment will be mediated by the student-perceived teacher autonomy support and reading achievement.

## Materials and methods

### Sample

This study used the secondary dataset from the PISA 2018, which was released in December 2019.^1^ As one of the most authoritative international large-scale datasets, the PISA evaluates how well 15-year-old students mastered the compulsory education knowledge of reading, mathematics, and science via real-life scenarios. Additionally, students’ ability to fully participate in modern society can be assessed in varieties of learning circumstances and academic settings. Conducted every 3 years, the PISA provides a comprehensive and rigorous benchmark measurement for national educational situations and allows comparisons across countries. In the PISA 2018, the major domain tested was reading achievement. This study analyzed a subset of PISA database, reading achievement, which comprises a total of 12,058 students from 361 schools in China with the proportions of 57.2% for males and 47.8% for females.

### Variables

#### Student-perceived teacher autonomy support

There were four items evaluating student-perceived teacher autonomy support in reading encouragement (e.g., “The teacher encourages students to express their opinion about a text,” see Supplementary materials). Adolescents were invited to respond to the questionnaire about their perception of teacher autonomy support with a four-point Likert scale ranging from “1 = Never or hardly ever” to “4 = In all lessons.” To measure of internal reliability, Cronbach’s alpha was adopted in this study. The internal reliability for teacher autonomy support is 0.90.

#### Control and value appraisals

##### Reading self-efficacy

This variable evaluates students’ self-concept of their competence in reading. This variable was measured by three items on a four-point Likert-type scale (e.g., “I am a good reader.”), ranging from “1 = strongly disagree” to “4 = strongly agree.” The internal reliability for reading self-efficacy is 0.794.

##### Meaning in life

This variable evaluates a sense of meaning and purpose that a student might have in terms of their experience. This variable was measured by three items (e.g., “My life has clear meaning or purpose.”) and adolescents were asked to complete a four-point Likert-scale questionnaire ranging from “strongly disagree,” to “strongly agree.” The internal reliability for meaning in life is 0.914.

#### Reading enjoyment

This variable evaluates students’ enjoyment of reading. This variable was measured by five items (e.g., “Reading is one of my favorite hobbies”) on a four-point Likert-type scale ranging from “1 = strongly disagree” to “4 = strongly agree.” Considering the negatively worded items, reverse-scored Item Response Theory (IRT) scaling was adopted. The internal reliability for reading enjoyment is 0.812.

#### Reading achievement

According to the PISA 2018 reading assessment, reading achievement is defined as being able to comprehend, analyze and engage with the texts, to build knowledge and accomplish their goals to effectively participant in society (OECD, 2019, p. 28). Specifically, PISA 2018 reading assessment provides correct estimates by addressing the cognitive processes of adolescents, including retrieving and synthesizing information, evaluating and reflecting from manifold text formats, and reading contexts (e.g., novels, personal letters). In the current study, the reading achievement was assessed using one of the plausible values (i.e., PV1 reading) grounded on the item response models (OECD, 2019). The results were normalized on a scale across OECD countries with a mean of 500 (SD = 100).

#### Control variables

Previous studies revealed that demographic variables (e.g., socioeconomic status, gender) exerted a substantial influence on adolescents’ reading achievement (e.g., Korhonen et al., 2016; Ma et al., 2018; Rogiers et al., 2020). Students with higher socioeconomic status (ESCS) tend to be much more proficient in reading than students with lower ESCS. Additionally, gender differences have been consistently revealed to have significant associations with the reading ability (Nalipay et al., 2019). Therefore, this study adopted ESCS and gender as demographic control variables. Student gender was coded as 1 for girls and 0 for boys and then transferred into dummy variables. ESCS was measured from a combination of three variables, including parents’ occupational status, parents’ educational level, and home possessions (OECD, 2021).

### Statistical models

This study applied a serial multiple mediation model to examine the relations between teacher autonomy support, control-value appraisals, achievement emotions and reading achievement. According to Preacher and Hayes (2008), multiple mediation model involves several mediators in one model and allows for analyzing the relative magnitudes of the direct and indirect correlations related to mediators. Compared to the simple mediation model with one mediator in several separate models, multiple mediation model can reduce the likelihood of parameter bias and provide reliable standard errors (Preacher and Hayes, 2008; Kenny, 2018).

The hypothesized multiple mediation model was tested using the lavaan package in R (R Core Team, 2020). As for the estimation method, the Maximum likelihood estimation with robust standard errors (MLR) was used. Additionally, model fit was estimated using the comparative fit index (CFI), Tucker-Lewis index (TLI), root mean square error of approximation (RMSEA), and standardized root means square residual (SRMR). Accordingly, the acceptable model fit index is that both the CFI and TLI are above 0.95, RMSEA below 0.08, and SRMR below 0.10 (Kline, 2015). Before conducting the outcome analysis, the missing data were imputed with the expectation-maximization (EM) algorithm across the entire dataset. All continuous variables were converted to the centralized data. It is also worth stressing that student weights were calculated among the measures examined.

## Results

Table 1 presents the mean and standard deviation of, and the associations between selected variables. Table 2 presents the results of the model fit. Based on the criteria proposed by Kline (2015), the CFI (0.99) and TLI (0.95) greater than 0.9, whereas the RMSEA and SRMR smaller than 0.08, indicating that the model fit was in an acceptable range.

**TABLE 1:** Descriptive statistics and correlation matrices among variables.

Variable	M	SD	1	2	3	4	5	6
GENDER	1.52	0.50	–					
ESCS	−0.36	1.08	−0.020*	–				
AUTOSUP	0.63	1.03	−0.072**	0.180**	–			
SCREADCOMP	0.08	0.86	0.049**	0.287**	0.298**	–		
EUDMO	0.09	0.91	−0.011	0.071**	0.255**	0.317**	–	
JOYREAD	0.98	0.84	−0.145**	0.201**	0.319**	0.595**	0.227**	–

*P < 0.05, **P < 0.01.

**TABLE 2:** Fit indices and the parameter estimates of the final model.

PISA 2018 reading assessment (China, *n* = 12,058)				
**Model fit**				
Metrics	RMSEA mean (SD)	0.078 (0.000)		
	CFI mean (SD)	0.990 (0.000)		
	TLI mean (SD)	0.947 (0.000)		
	SRMR mean (SD)	0.045 (0.000)		
**Direct effects**		*b*	*SE*	*p*
AUTOSUP	→ APPRAISALS	0.257	0.009	0.000***
	→ JOYREAD	0.051	0.012	0.000***
	→ PVREAD	2.790	1.120	0.0131*
Appraisals	→ PVREAD	−8.870	3.360	0.008**
	→ JOYREAD	0.864	0.031	0.000***
JOYREAD	→ PVREAD	32.700	1.970	0.000***
**Indirect effects**				
AUTOSUP	→ APPRAISALS → PVREAD	−2.280	0.868	0.009**
	→ JOYREAD→ PVREAD	1.660	0.387	0.000***
	→ APPRAISALS → JOYREAD	0.222	0.012	0.000***
	→ APPRAISALS → JOYREAD → PVREAD	7.270	0.655	0.000***
**Total effects**				
AUTOSUP	PVREAD	9.430	1.1	0.000***
**Control variables**				
GENDER	→ APPRAISALS	0.021	0.017	0.212
	→ JOYREAD	−0.262	0.016	0.000***
	→ PVREAD	1.650	1.720	0.339
ESCS	→ APPRAISALS	0.237	0.012	0.000***
	→ JOYREAD	−0.046	0.012	0.000***
	→ PVREAD	14.400	1.430	0.000***

CFI refers to the comparative fit index, TLI refers to the Tucker-Lewis index, RMESA refers to the root mean square error of approximation, and SRMR refers to the standardized root means square residual; *P < 0.05, **P < 0.01, ***P < 0.001.

**FIGURE 2:**
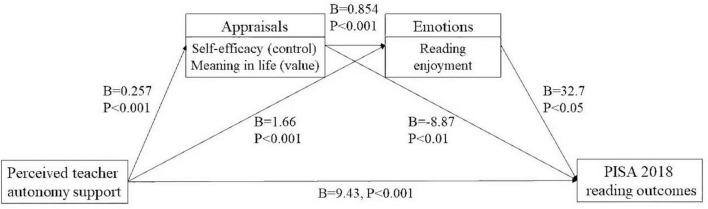
Summary of the full model in this study.

As Table 2 shown, student-perceived teacher autonomy support is positively associated with students’ reading achievement after controlling for students’ ESCS and gender (*B* = 2.79, SE = 1.12, *P* < 0.05), which indicates that the more students believe teacher autonomy support provided, the higher the score of their reading achievement. Meanwhile, student-perceived teacher autonomy support is significantly positively linked to control-value appraisals, i.e., self-efficacy and meaning in life (*B* = 0.257, SE = 0.009, *P* < 0.001), whereas appraisals are negatively linked to reading performance (*B* = −8.870, SE = 3.36, *P* < 0.05). Therefore, the control and value appraisals-related constructs are significantly and negatively mediated the relationship between student-perceived teacher autonomy support and reading performance (*B* = −2.280, SE = 0.868, *P* < 0.05). The results also indicate that reading enjoyment is significantly positively mediates the links between student-perceived teacher autonomy support and reading achievement (*B* = 1.660, SE = 0.387, *P* < 0.001). Additionally, Table 2 indicates that student-perceived teacher autonomy support is significantly positively associated with appraisals, i.e., self-efficacy and meaning in life, and have significantly positive relations to subsequent emotions, i.e., reading enjoyment (*B* = 0.222, SE = 0.012, *P* < 0.001). It elucidates that control- and value- related appraisals and reading enjoyment serially significantly positively mediates the correlation between student-perceived teacher autonomy support and reading achievement (*B* = 7.270, SE = 0.655, *P* < 0.001). Figure 2 presents a summary of the detailed model in this study.

## Discussion

This study extends the previous literature by identifying the relations among student-perceived teacher autonomy support, control and value appraisals, achievement emotions, and reading performance. The findings revealed that adolescents with higher perceived control and value in the reading assessment are more likely to gain more enjoyment and a real sense of fulfillment in the reading process, and subsequently attain higher reading outcomes. Moreover, based on CVT (Pekrun, 2006), the mediating role of appraisals (i.e., self-efficacy, meaning in life) and achievement emotions (i.e., enjoyment) in the associations between student-perceived teacher autonomy support and reading performance was examined. This study is of pivotal practical significance to CVT. First, the multiple mediation model adopted in this study provides additional evidence addressing the benefits of CVT on reading achievement of adolescents. Second, given that there is little empirical evidence that has thoroughly researched the interrelations of teacher autonomy support and reading performance under the framework of CVT, this study provides new findings by adopting an authoritative large-scale PISA-empirical-based evaluation in the Chinese context.

### The mediating role of control-value appraisals between student-perceived teacher autonomy support and reading achievement

Our results indicate that control and value appraisals are mediated by student-perceived teacher autonomy support and reading achievement. Although some studies have begun looking at the control-value appraisals on educational achievement, this study is the first to specifically examine the mediating role of appraisals (i.e., self-efficacy and meaning in life) between teacher autonomy support and reading achievement among secondary school students. Therefore, our results add to the literature by addressing that teacher autonomy support might enhance adolescents’ appraisals (i.e., reading self-efficacy and meaning in life) in the reading process, although the improvement of their self-efficacy and intrinsic values might not be positively related to reading achievement in the Chinese context.

Specifically, this finding reveals that control-value appraisals (i.e., self-efficacy and meaning in life) are negatively related to adolescents’ reading achievement, which is contradictory with a study conducted by Bailey and Phillips (2016). They hold the idea that intrinsic motivation (e.g., self-efficacy) and meaning in life are positively related to students’ academic performance in college. However, this result partly aligns with that of Carroll and Fox (2017), who demonstrated that reading self-efficacy is associated with word reading, but is not linked to reading comprehension. Additionally, as Schunk (1996) claimed that high self-efficacy without the necessary knowledge and abilities does not result in improved literacy, and can result in poor reading performance. These contrary perspectives can be explained as Eccles and Wigfield (2020) demonstrated that student self-concept and intrinsic value were complicated areas and should be situated into contextual factors. Pekrun (2006) and Peura et al. (2019b) further clarified that the differed results might be caused by general-domain and specific-domain ways of control- and value-related constructs.

Furthermore, this study indicates student-perceived teacher autonomy support is significantly positively related to the control-value appraisals, which partly aligns with a study conducted by Zimmermann et al. (2021) that mentioned a positive relationship between teacher autonomy support and control-value appraisals based on leisure physical activity. One potential underlying reason is that teacher autonomy support such as knowledge activation and stimulation for engagements make students be noticed in managing their learning processes; consequently, students might develop their self-efficacy and intrinsic values (Eccles, 2005; Peura et al., 2019a). Therefore, this study might provide an efficient motivating teaching style for educators who seek to cultivate students’ reading competence by enhancing their motivational beliefs, and encouraging their engagement in specific reading activities.

### The mediating role of reading enjoyment between student-perceived teacher autonomy support and reading achievement

Our findings suggest that the enjoyment of reading, the core component in achievement emotions mediates the links between student-perceived teacher autonomy support and reading performance, which extends the findings of previous studies that considered only the relationship between reading enjoyment and reading achievement. Essentially, this study provides a robust examination of the correlation between enjoyment in reading and reading performance. This study indicates a positive relation between reading enjoyment and reading performance, and dovetails with previous literature (e.g., Rogiers et al., 2020; Zaccoletti et al., 2020; Hu and Wang, 2022), which speculated that reading enjoyment can function as an essential psychological pathway to improvements in a reader’s reading fluency and knowledge base (Preece and Levy, 2020; Simonton and Garn, 2020). One possible reason might be that students who enjoy their reading class or assessments are more likely to be cognitively engaged with their courses (Xiao et al., 2019; Mercan, 2020), understand and use more contributory reading strategies, gain a more efficient interpretation of texts, and thus can achieve higher reading achievement (Ryan and Deci, 2020; Chen and Hu, 2021).

This study also suggests that student-perceived teacher autonomy support is significantly positively related to students’ enjoyment of reading. According to CVT theory, students’ achievement emotions can be developed in positive interactions with the learning environment and a host of learning activities (Pekrun and Stephens, 2010; Van der Beek et al., 2017). Thus, teacher autonomy support can satisfy adolescents’ mental requirements of relatedness, competence, and autonomy (Ryan and Deci, 2017; Moè and Katz, 2020). However, few studies have examined the relationship between teacher autonomy support, reading enjoyment, and reading literacy, and most of them are conducted among primary school students or college students, with little empirical evidence for secondary school students. Therefore, these current findings add to the literature by addressing the positive associations of teacher autonomy support with students’ reading enjoyment and reading performance. However, due to the lack of information about students’ negative emotions (e.g., depression, nervousness) in reading process, the variations between different emotional factors call for further empirical studies; future studies could elucidate whether teacher autonomy support is beneficial for reducing the negative emotions and enhancing student wellbeing.

### Control-value appraisals as the antecedent of reading enjoyment

Our findings indicate that the control-value appraisals (i.e., reading self-efficacy, meaning in life) and then subsequent emotions (i.e., reading enjoyment) are positively mediates the relations between teacher autonomy support and reading attainment. In other words, reading self-efficacy and meaning in life are positively related to reading achievement through reading enjoyment. The likely mechanism is that students who have strong motivational beliefs and intrinsic values are more likely to persevere in and overcome potential difficulties, so they tend to have positive emotions in the reading process, i.e., underpin enjoyment in reading, and thus enhance their reading achievement. This result resonates with studies conducted by Pekrun (2006) and Simonton and Garn (2020), which demonstrated that control-value appraisals serves as an antecedent of achievement emotions, such as enjoyment or boredom. Likewise, in a longitudinal study, Putwain et al. (2021) reported that students’ perception of their competence and intrinsic value can amplify their enjoyment, and improve their math achievement. However, this result is contradictory to a study conducted by Guay et al. (2019) that elucidated an opposite direction of the influence pathway. That is, students’ self-perception of competence mediates the links between enjoyment and reading achievement. This empirical evidence is varied due to different subjects and different evaluations of assessments (e.g., Peura et al., 2019b). Additionally, different samples adopted in studies also cause different results. That is, Guay et al. (2019) focused on primary school students, while this study investigated secondary school students. From the viewpoint of individual development, children of a young age tend to show interest in reading, thus influencing their self-perception of competence, whereas secondary school adolescents might show a differential direction between self-efficacy and reading enjoyment as they grow up (Ma et al., 2018; Westphal et al., 2018). Therefore, it is essential to emphasize their relationship direction, the antecedent role of the control value, i.e., self-efficacy and meaning in life, in their emotions considering different age groups.

Since the influential patterns of teacher autonomy support on control and value constructs and emotional factors was unknown, further studies could make a comparison of their relationship at different growth stages and identify a pathway through a series of developmental stages.

## Conclusion

This study contributes to the previous literature by examining a complicated relationship between teacher autonomy support, control-value appraisals (i.e., self-efficacy, meaning in life), achievement emotions (i.e., reading enjoyment), and reading achievement among 15-year-old adolescents drawing on the sub-dataset of China in the PISA 2018 assessment. To our best knowledge, this is one of the first empirical studies in the educational field to examine the mediation role of control and value appraisals (i.e., reading self-efficacy and meaning in life), achievement emotions (i.e., reading enjoyment) playing between teacher autonomy support and reading achievement based on the control-value theory. Despite the relations between teacher autonomy support and educational attainment has been examined, the mechanism behind them, i.e., how teacher autonomy support works in specific academic settings have not been thoroughly explored. This study yielded three new viewpoints. First, this study offers a comprehensive perspective and probes into the reading classroom by revealing a positive relationship among teacher autonomy support, control-value appraisals, i.e., self-efficacy, meaning in life, and reading enjoyment. Second, this study found that the antecedent role of control and value appraisals in reading enjoyment, that is, the positive influence of self-efficacy and meaning in life on reading enjoyment, suggesting the direction of this relation should be taken into account. Third, since most of the previous studies examined the relationship between teacher autonomy support and reading achievement among primary school students or college students, this study adds to the previous literature by addressing 15-year-old adolescents from a large-scale and authoritative PISA 2018 data set. These findings have crucial implications for researchers, teachers and school policymakers seeking alternative solutions and multiple paths to puzzling adolescents’ various learning problems, and provide guidance for teachers to fully utilize the cognitive activation and stimulated strategies to enhance students’ different motivational beliefs and provoke their positive emotions, which contribute a lot on students’ reading achievement.

## Limitations and implications

It is acknowledged that this study has several limitations to highlight. First, with respect to the data variation in the cross-sectional study, the selected sample is limited. Future empirical studies can consider the longitudinal data to detect the dynamic individual development and further elucidate this cause-and-effect correlation. Second, since most of the PISA assessment questionnaires were self-reported by adolescents, it might cause some endogeneity bias. Therefore, it is beneficial to include other measurements such as in-depth observation, the teacher-reported data when assessing teacher autonomy support, to offer more detailed explanations for readers. Third, our test of the correlations of appraisals and achievement emotions was limited to perceived control, intrinsic value and positive emotions due to the characteristics of multiple mediation modeling, other variables like extrinsic value, and negative emotions could be studied in further studies.

## Data availability statement

The original contributions presented in this study are included in the article/Supplementary material, further inquiries can be directed to the corresponding author.

## Ethics statement

The utilization of the sample, data, and data analysis procedure of the present study has been approved by the Medical Ethics Committee of Department of Psychology and Behavioral Sciences, Zhejiang University [ethics approval number: (2021) No. 74] and the authors declare no ethnical violation. Written informed consent to participate in this study was provided by the participants’ legal guardian/next of kin. Written informed consent was obtained from the individual(s), and minor(s)’ legal guardian/next of kin, for the publication of any potentially identifiable images or data included in this article.

## Author contributions

MW: conceptualization, methodology, data analysis, writing, and editing. JH: supervision, methodology, data analysis, writing, reviewing, and editing. Both authors contributed to the article and approved the submitted version.

## Footnotes

1
https://www.oecd.org/pisa/data/2018database/
