# Supplementary material for: Perceived teacher autonomy support for adolescents’ reading achievement: The mediation roles of control-value appraisals and emotions
Source: Front Psychol. 2022 Aug 29;13:959461. doi: 10.3389/fpsyg.2022.959461 (PMC9466293; doi:10.3389/fpsyg.2022.959461)
Supplement: Supplementary file 1 [file Table_1.docx]

**Appendix A.** Questionnaire items corresponding to the main variables.

| **Category** | **Variable ID** | **Description** | **Value Scale** |
| --- | --- | --- | --- |
| Student perceived teacher autonomy support | ST152Q05IA | The teacher encourages students to express their opinion about a text. | 1=Never or hardly ever,  2=In some lessons,  3=In most lessons,  4=In all lessons. |
|  | ST152Q06IA | The teacher helps students relate the stories they read to their lives. | Same as above |
|  | ST152Q07IA | The teacher shows students how the information in texts builds on what they already know. | Same as above |
|  | ST152Q08IA | The teacher poses questions that motivate students to participate actively. | Same as above |
| Perceived control  Self-concept of reading: Perception of competence (WLE) | ST161Q01HA | I am a good reader. | 1 = Strongly disagree,  2 = Disagree,  3 = Agree,  4 = Strongly agree. |
|  | ST161Q02HA | I am able to understand difficult texts. | Same as above |
|  | ST161Q03HA | I read fluently. | Same as above |
| Intrinsic value  Eudaemonia: meaning in life  (WLE) | ST185Q01HA | My life has clear meaning or purpose. | 1 = Never,  2 = Rarely,  3 = Sometimes,  4 = Always. |
|  | ST185Q02HA | I have discovered a satisfactory meaning  in life. | Same as above |
|  | ST185Q03HA | I have a clear sense of what gives  meaning to my life. | Same as above |
| Positive emotions  (Reading enjoyment ) | ST160Q01IA | I read only if I have to. | 1 = Strongly disagree,  2 = Disagree,  3 = Agree,  4 = Strongly agree. |
|  | ST160Q02IA | Reading is one of my favorite hobbies. | Same as above |
|  | ST160Q03IA | I like talking about books with other people. | Same as above |
|  | ST160Q04IA | For me, reading is a waste of time. | Same as above |
|  | ST160Q05IA | I read only to get information that I need. | Same as above |

**Appendix B.**VIFs for all selected variables

| **Variable** | **PISA 2018** |
| --- | --- |
| Gender | 1.000 |
| ESCS | 1.43 |
| STIMREAD | 2.06 |
| SCREADCOMP | 2.64 |
| EUDMO | 3.94 |
| JOYREAD | 2.36 |
